# ASYMPTOMATIC CHOLELITHIASIS: EXPECTANT OR CHOLECYSTECTOMY. A SYSTEMATIC REVIEW

**DOI:** 10.1590/0102-672020230029e1747

**Published:** 2023-07-17

**Authors:** Jose Roberto Alves, Diurlhane Mainara Klock, Filipe Gonçalves Ronzani, Sheyne Luiz dos Santos, Enio Campos Amico

**Affiliations:** 1 Universidade Federal de Santa Catarina, Department of Surgery – Florianópolis (SC), Brazil; 2 Universidade Federal de Santa Catarina, School of Medicine – Florianópolis (SC), Brazil; 3 Hospital Universitário Polydoro Ernani de São Thiago, General Surgery – Florianópolis (SC), Brazil; 4 Universidade Federal do Rio Grande do Norte, Department of Integrated Medicine – Natal (RN), Brazil.

**Keywords:** Cholecystectomy, Cholelithiasis, Gallstones, Gallbladder, Colecistectomia, Colelitíase, Cálculos biliares, Vesícula biliar

## Abstract

**BACKGROUND:**

Asymptomatic cholelithiasis is a highly prevalent disease, and became more evident after the currently greater access to imaging tests. Therefore, it is increasingly necessary to analyse the risks and benefits of performing a prophylactic cholecystectomy.

**AIMS:**

To seek the best evidence in order to indicate prophylactic cholecystectomy or conservative treatment (clinical follow-up) in patients with asymptomatic cholelithiasis.

**METHODS:**

A systematic review was performed using the PubMed/Medline database, according to PRISMA protocol guidelines. The review was based on studies published between April 26, 2001 and January 07, 2022, related to individuals older than 18 years., The following terms/operators were used for search standardization: (asymptomatic OR silent) AND (gallstones OR cholelithiasis).

**RESULTS:**

We selected 18 studies eligible for inference production after applying the inclusion and exclusion criteria. Also, the *Tokyo Guideline* (2018) was included for better clarification of some topics less or not addressed in these studies.

**CONCLUSIONS:**

Most evidence point to the safety and feasibility of conservative treatment (clinical follow-up) of asymptomatic cholelithiasis. However, in post-cardiac transplant patients and those with biliary microlithiasis with low preoperative surgical risk, a prophylactic cholecystectomy is recommended. To establish these recommendations, more studies with better levels of evidence must be conducted.

## RESUMO

**RACIONAL:**

A colelitíase assintomática é uma doença altamente prevalente, e atualmente tornou-se mais evidente, após o maior acesso a exames de imagem. Portanto, é cada vez mais necessário analisar os riscos e benefícios de realizar uma colecistectomia profilática.

**OBJETIVOS:**

Buscar as melhores evidências para indicar colecistectomia profilática ou tratamento conservador (acompanhamento clínico) em pacientes com colelitíase assintomática.

**MÉTODOS:**

Foi realizada revisão sistemática, no PubMed/Medline, de acordo com as diretrizes do protocolo *PRISMA*, selecionando estudos publicados entre 26/04/2001 e 01/07/2022, relacionados a indivíduos maiores de 18 anos, com padronização de busca usando os seguintes termos/operadores: (Assintomático OU Silencioso) E (Cálculos biliares OU colelitíase).

**RESULTADOS:**

Foram selecionados 18 estudos elegíveis após a aplicação dos critérios de inclusão e exclusão. Além disso, a *Tokyo Guideline* (2018) foi incluída para melhor esclarecimento de alguns tópicos menos ou não abordados nestes estudos.

**CONCLUSÕES:**

A maioria das evidências aponta para a segurança e viabilidade do tratamento conservador (acompanhamento clínico) da colelitíase assintomática. Entretanto, em pacientes com transplante pós-cardíaco e aqueles com microlitíase biliar com baixo risco cirúrgico pré-operatório, a colecistectomia profilática é recomendada. Para estabelecer completamente estas recomendações, mais estudos com melhores níveis de evidência devem ser conduzidos.

## INTRODUCTION

Cholelithiasis or calculous cholecystopathy is a highly prevalent disease that affects up to 15% of the adult world population^1,6,12,20,23^. However, its incidence varies according to age, gender, ethnicity, diet, geography, socioeconomic conditions, comorbidities, and other coexisting clinical conditions^1,6,12,14,19,20,23^. In some special situations, the incidence rates are higher, reaching 53% in post-bariatric surgery and 54% in cirrhotic patients^2,20,23^. Nowadays, the diagnosis of cholelithiasis frequently occurs incidentally, given the increased accessibility and higher number of requests for ultrasonographic examination of the abdomen, during the investigation of several causes^11,16^.

About 1 to 4% of asymptomatic cholelithiasis (AC) cases become symptomatic each year, with a consequent risk of about 20% over 20 years of follow-up^21^. Among the potential complications are pain of biliary origin (biliary colic), acute cholecystitis, gallbladder empyema, cholangitis, acute pancreatitis^1,15,22^. It is valid to describe that pain of biliary origin has characteristics of colicky abdominal pain, located in the epigastric or right hypochondrium or both, in general, lasting more than 30 minutes; it may or may not start after a fatty meal and does not relieve with antacids, with the possibility to irradiate to the ipsilateral dorsal region, the inferior portion of the scapula, right shoulder, or a combination of them, associated, in general, with nausea and, occasionally, vomiting^3,6,11^. However, when acute cholecystitis is present, the pain is typically localized in the right hypochondrium, with a longer duration, associated with positive Murphy's sign and fever^11^. Patients may also present with signs of cholestasis (as in cases of choledocholithiasis and cholangitis), more intense epigastralgia (particularly in cases of pancreatitis), or sepsis^11^.

Some pathologies increase the chance of AC carriers becoming symptomatic at some point, for example, in those patients with coronary artery disease, metabolic syndrome, sickle cell anemia, obesity, and patients who have lost weight too quickly^14,15^.

Except for high surgical risk, patients with symptomatic cholelithiasis or who present some complication related to gallstones have laparoscopic cholecystectomy as their most appropriate therapeutic option. However, there is still no formal consensus on the indication of prophylactic cholecystectomy in asymptomatic cases. Classically, there is a tendency to indicate prophylactic cholecystectomy in younger patients with AC, carriers of larger stones (>2.5–3 cm), gallbladder polyps >1 cm, biliary microlithiasis, sickle cell anemia, dysfunctional gallbladder or with calcified walls (porcelain gallbladder)^6,11,13,21^. It should also be considered, especially if these conditions are associated with potential risk factors for gallbladder cancer (age ≥65 years, jaundice, women, raised alkaline phosphatase, focal gallbladder wall thickening ≥5 mm, biliopancreatic maljunction, and a dilated bile duct)^7^.

When prophylactic cholecystectomy is indicated, it is crucial to consider the possible complications inherent to the surgical and anesthetic procedure and post-cholecystectomy complications^1^. It is also known that the onset or persistence of abdominal pain or gastrointestinal symptoms may occur in a considerable percentage of post-cholecystectomy patients^1^.

Thus, cholecystectomy in asymptomatic patients remains a controversial issue. Considering this and based on a systematic review, which includes studies from the last 21 years, we aimed to present the most appropriate indications for prophylactic cholecystectomy in patients with AC and also to better evaluate the potential consequences of the indication for this procedure.

## METHODS

A systematic review was carried out utilizing the PubMed/Medline, from April 26, 2001 to January 07, 2022, according to the recommendations of the PRISMA protocol (Preferred Reporting Items for Systematic Reviews and Meta-Analysis)^18^. This search included studies published in English and Portuguese on clinical studies, clinical trials, clinical trial protocols, clinical trials in phase IV, comparative studies, controlled clinical trials, meta-analysis, multicenter studies, and randomized controlled trials. The following search strategy (selection of terms/Boolean operators) was used: (asymptomatic OR silent) AND (gallstones OR cholelithiasis). Studies with limited access and those involving patients younger than 18 years old were excluded.

The search results from PubMed/Medline were transferred to the Rayyan platform^13^ to facilitate the selection of eligible studies and to exclude duplicate/triplicate publications. At least, two independent authors read all abstracts and applied the inclusion and exclusion criteria. Afterwards, they read the selected studies in their entirety, in order to produce the inferences presented below. The selected studies were classified according to the level of evidence from I to V and the degree of recommendation from A to D, according to the Oxford Centre for Evidence-Based Medicine (Table 1)^17^.

**Table 1:** Classification of references according to the level of evidence and degree of recommendation according to the Oxford Centre for Evidence-Based Medicine^17^

Level of evidence and Degree of recommendation	Studies encompassed
1A	Systematic review of randomized clinical trials; Systematic review of prospective cohorts; Prognostic criteria validated in several populations; Systematic review of level 1 diagnostic studies.
1B	Randomized controlled clinical trials with narrow confidence interval; Cohort from disease onset, with loss <20%; Validated prognostic criteria in a single population; Validated cohort with a good baseline.
1C	All-or-nothing therapeutic results.
2A	Systematic review of cohort studies; Systematic review of retrospective or follow-up cohorts of untreated cases from a randomized clinical trial control group; Systematic review of diagnostic studies level >2.
2B	Cohort study (including randomized clinical trial of lower quality; e.g., <80% follow-up); Retrospective cohort study or follow-up of untreated control patients in randomized clinical trials.
2C	Observation of therapeutic outcomes (outcomes research); Ecological study.
3A	Systematic review of case-control studies; Systematic review of diagnostic studies level >3B.
3B	Case-control study; Non-consecutive study or no consistently applied benchmarks; Non-consecutive cohort study or very limited population.
4	Case report; Case series; Low-quality cohort and case-control studies.
5	Expert opinion without explicit critical appraisal or based on physiology, bench research, or “first principles”.

For the clinical trials, the Jadad scale was also applied to better express the methodological quality of each one of them (Table 2)^13^.

**Table 2:** Classification of clinical trials according to quality criteria and scoring according to the Jadad scale^13^.

Studies	Presence of Randomization	Appropriate randomization method	Study was double-blind	Appropriate double-blind method	Sample Loss Description	Total
Ahmed et al.^1^	+1	+1	0	0	+1	+3
Bencini et al.^2^	+1	+1	+1	+1	+1	+5
Habeeb et al.^9^	+1	0	0	0	+1	+2
Hyun et al.^11^	0	0	0	0	+1	+1

## RESULTS

After searching the Pubmed/Medline database according to the predefined inclusion criteria, 70 studies were initially identified and sent to the Rayyan platform. No duplicate/triplicate studies were found, and after applying the exclusion criteria, 47 studies were excluded due to study population (n=26) or study outcome (n=21) outside the area of interest of this review.

Thus, 23 studies were obtained and evaluated by the authors through a complete reading of their content, and subsequently five other studies were excluded.

**Figure 1:**
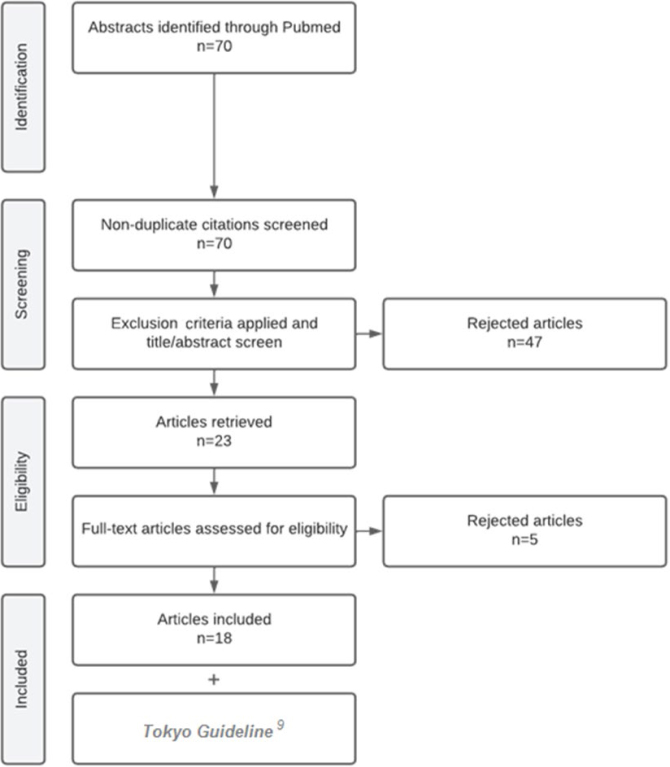
Flowchart demonstrating the systematization and selection of eligible studies for inference production.

Finally, 18 studies were selected for this review (Figure 1). In addition, the Tokyo Guideline (2018)^9^ was included to better clarify the minor or not addressed points in these eligible studies. The PRISMA protocol was confirmed through the checklist illustrated in Table 3 ^17,18^.

**Table 3:** PRISMA Checklist.

Section/topic		Checklist item	Reported on page #
TITLE			
	Title	Identify the report as a systematic review, meta-analysis, or both.	1
ABSTRACT			
	Structured summary	Provide a structured summary including, as applicable: background; objectives; data sources; study eligibility criteria, participants, and interventions; study appraisal and synthesis methods; results; limitations; conclusions and implications of key findings; systematic review registration number.	1
INTRODUCTION			
	Rationale	Describe the rationale for the review in the context of what is already known.	1–2
	Objectives	Provide an explicit statement of questions being addressed with reference to PICOS.	2
METHODS			
	Protocol and registration	Indicate if a review protocol exists, if and where it can be accessed (e.g., web address), and, if available, provide registration information including registration number.	N/A
	Eligibility criteria	Specify study characteristics (e.g., PICOS, length of follow-up) and report characteristics (e.g., years considered, language, publication status) used as criteria for eligibility, giving rationale.	3
	Information sources	Describe all information sources (e.g., databases with dates of coverage, contact with study authors to identify additional studies) in the search and date last searched.	3
	Search	Present full electronic search strategy for at least one database, including any limits used, such that it could be repeated.	3
	Study selection	State the process for selecting studies (i.e., screening, eligibility, included in systematic review, and, if applicable, included in the meta-analysis).	3
	Data collection process	Describe method of data extraction from reports (e.g., piloted forms, independently, in duplicate) and any processes for obtaining and confirming data from investigators.	3
	Data items	List and define all variables for which data were sought (e.g., PICOS, funding sources) and any assumptions and simplifications made.	N/A
	Risk of bias in individual studies	Describe methods used for assessing risk of bias of individual studies (including specification of whether this was done at the study or outcome level), and how this information is to be used in any data synthesis.	N/A
	Summary measures	State the principal summary measures (e.g., risk ratio, difference in means).	2–3
	Synthesis of results	Describe the methods of handling data and combining results of studies, if done, including measures of consistency (e.g., I2) for each meta-analysis.	3
	Risk of bias across studies	Specify any assessment of risk of bias that may affect the cumulative evidence (e.g., publication bias, selective reporting within studies).	N/A
	Additional analyses	Describe methods of additional analyses (e.g., sensitivity or subgroup analyses, meta-regression), if done, indicating which were pre-specified.	N/A
RESULTS			
	Study selection	Give numbers of studies screened, assessed for eligibility, and included in the review, with reasons for exclusions at each stage, ideally with a flow diagram.	3
	Study characteristics	For each study, present characteristics for which data were extracted (e.g., study size, PICOS, follow-up period) and provide the citations.	N/A
	Risk of bias within studies	Present data on risk of bias of each study and, if available, any outcome level assessment (see item 12).	3–8
	Results of individual studies	For all outcomes considered (benefits or harms), present, for each study: (a) simple summary data for each intervention group (b) effect estimates and confidence intervals, ideally with a forest plot.	N/A
	Synthesis of results	Present results of each meta-analysis done, including confidence intervals and measures of consistency.	N/A
	Risk of bias across studies	Present results of any assessment of risk of bias across studies (see Item 15).	N/A
	Additional analysis	Give results of additional analyses, if done (e.g., sensitivity or subgroup analyses, meta-regression [see Item 16]).	N/A
DISCUSSION			
	Summary of evidence	Summarize the main findings including the strength of evidence for each main outcome; consider their relevance to key groups (e.g., healthcare providers, users, and policy makers).	3–8
	Limitations	Discuss limitations at study and outcome level (e.g., risk of bias), and at review-level (e.g., incomplete retrieval of identified research, reporting bias).	3–8
	Conclusions	Provide a general interpretation of the results in the context of other evidence, and implications for future research.	3–8
FUNDING			
	Funding	Describe sources of funding for the systematic review and other support (e.g., supply of data); role of funders for the systematic review.	8

PICOS: participants, interventions, comparisons, outcomes, and study design.

## DISCUSSION

In patients with AC, clinical follow-up shows a benign clinical course (level of evidence: IB)^1,3,6,16^. Overall, patients with AC have an annual chance of developing some related symptom or complication in 1–4% of cases at longer follow-up (mean 8.7 years) (IB)^1,6^. However, it is important to be aware that before any AC-related complication (acute cholecystitis, cholangitis, and acute pancreatitis) occurs, it will almost always be preceded by biliary pain or some milder related clinical manifestation (IIB)^21^. In addition, more than half of cases that become symptomatic will not have more than one clinical episode, and the severity of the disease will not increase over time^1,6,14^. Furthermore, even if the patient with AC has a symptomatic episode, there is a 58.5% chance in mild cases and a 52.1% in moderate cases that this symptom will not appear again in a long follow-up period (mean of 8.7 years) (IIB)^6^. In a study with a 10-year follow-up of non-operated cases of AC, only 22% developed clinical manifestations or complications related to the presence of gallstones^6^. Finally, we must consider that aging may be associated with a higher risk of symptoms (IIB)^6^.

Furthermore, it should be known that the conservative therapeutic plan, based on clinical follow-up only, is a lower cost option for public health when compared to prophylactic cholecystectomy performed in patients with AC, which is an essential factor to be considered since calculous cholecystopathy is currently considered the digestive tract disease related to the highest hospital costs involving inpatient medical services (IB)^1,6^.

Another factor worth noting, which is clearly documented, is that the risk of developing cancer in patients with gallstones is less than 0.01%, i.e., less than the mortality associated with performing a cholecystectomy^6^. In addition, when deciding to perform cholecystectomy, despite conflicting results, one should take into account and recognize a possible higher risk of developing colon cancer at long-term follow-up after cholecystectomy^6^.

Still, conservative management may present benefits even in certain clinical conditions with an increased incidence of cholelithiasis compared to the general population. For example, up to 25% of patients undergoing gastrectomy may develop cholelithiasis (due to injury to the vagus nerve branches and anatomical changes inherent to the surgery)^2^. In these patients, although prophylactic cholecystectomy performed concomitantly with gastrectomy (for malignant neoplasms) reduces the incidence of future gallbladder abnormalities and does not generate considerable additional intraoperative time, there is no significant impact on the clinical follow-up of these patients^2^. Thus, prophylactic gallbladder removal in these patients is not warranted at this time if the only concern is a late development of cholelithiasis (IB)-related symptoms and complications^2^. Further studies are needed, however, to ascertain whether this inference applies to all patients who undergo gastrectomy or whether it is restricted only to those who have had surgery to treat gastric cancers^2^.

Another example is patients with liver cirrhosis. It is known that these patients are 1.2 to 3 times more likely to present cholelithiasis than the general population, appearing as an incidental finding on ultrasound abdomen examinations in up to 54% of cirrhotic patients^5^. The more severe their existing liver disease (level of evidence VD) is, cholelithiasis is even more prevalent^23^. It is observed that AC is adequately managed in a conservative form in cirrhotic patients, provided close and rigorous follow-up is performed to detect early possible symptoms and complications (VD)^23^. However, this is low evidence, once it is related to an old publication (2005), requiring other better-designed studies (double-blind, randomized) for the definitive establishment of this recommendation^23^. An inherent disadvantage of conservative treatment is that if the cholelithiasis is no longer asymptomatic in these patients with chronic liver disease, then the morbidity and mortality related to cholecystectomy will be higher compared to non-cirrhotic patients (VD)^23^. However, surgery and general anesthesia are considered risky in this patient population. Performing a laparoscopic cholecystectomy involves an overall morbidity rate of 21% in cirrhotic patients compared to 8% morbidity in non-cirrhotic patients^23^. Given the above and the existing evidence to date, it is generally recommended that patients with concomitant AC and liver cirrhosis can be managed conservatively but under close medical monitoring, aiming at early detection of possible clinical changes (VD)^23,25^.

Another group of patients worth considering regarding the need to perform prophylactic cholecystectomy is those who will undergo organ transplants. In liver transplantation cases, cholecystectomy is an inherent part of the procedure due to the removal of the adjacent organ. However, in kidney, pancreatic, and heart transplants, there are other factors to be evaluated. First, it is identified that patients undergoing kidney transplantation (IIB)^12^ or pancreas transplantation (IIIA)^4^ have the same incidence of AC, the same rates of conversion of AC to symptomatic cases, and the occurrence of related complications as the general population^4,12^. Therefore, similarly, although cholecystectomy in an emergency is associated with higher morbidity and mortality, the risks associated with the clinical follow-up (conservative) of AC do not seem to justify prophylactic cholecystectomy in patients waiting for kidney transplantation (IIB) or pancreatic transplantation (IIIA)^4,12^. In cardiac transplant patients, however, the current evidence is different. There are fewer proportional deaths in those heart transplant patients who undergo prophylactic cholecystectomy after transplantation (5:1000) compared to conservative follow-up (44:1000) and prophylactic cholecystectomy before transplantation (80:1000) (IIIA)^4^. In addition, a study reported that performing prophylactic cholecystectomy after heart transplantation resulted in cost savings of more than U$ 17,779 when evaluated by the *quality-adjusted life-year* questionnaire (IIIA)^4^. Thus, performing prophylactic cholecystectomy after transplantation is a recommended strategy in heart transplant patients with AC (IIIA)^4^, while those listed for kidney (IIB)^12^ and pancreatic (IIIA) transplantation^4^ should be conducted similarly to the general population.

We must address three other issues that may generate some controversy. The first is the indication of prophylactic cholecystectomy in patients with gallbladder polyps concomitant with AC. Although both are relatively common gallbladder abnormalities, AC and gallbladder polyps rarely coexist^8^. It is conjectured that this fact is justified due to the ultrasonographic difficulty in distinguishing these abnormalities or due to a possible destructive mechanical effect of stone movement on polyps (IIIB)^8^. Also, it is observed that the natural history of patients with gallbladder polyps concomitant with AC does not differ from those with polyps alone, so it is sparingly recommended that asymptomatic patients with gallbladder polyps and cholelithiasis should not be candidates for prophylactic cholelithiasis but should be followed, strictly, with serial abdominal ultrasound every three to six months (IIIB). Cholecystectomy is indicated only in indirect signs of malignancy related to these polyps (thickened, irregular gallbladder wall, increased polyp size during follow-up, polyps >1 cm)^6,8^.

The second issue concerns the incidental presence of biliary microlithiasis (gallstones smaller than 4 mm, usually not visible on abdominal ultrasound or cholecystography) as to the indication of prophylactic cholecystectomy^22^. It is known that microlithiasis can cause all possible manifestations and complications of cholelithiasis, particularly acute pancreatitis^22^. In a case-control study, microlithiasis was identified in 75% of patients with idiopathic acute pancreatitis and 83.3% with unexplained biliary pain (IVC)^22^. Therapeutic options for microlithiasis, depending mainly on the preoperative surgical risk related to each patient, include cholecystectomy, endoscopic sphincterotomy, and chemical dissolution through ursodeoxycholic acid (in older patients and those with high surgical risk^22,24^. A study stated that, with these therapies, there was a significant decrease in the recurrence rate (<10%) of ongoing pancreatitis episodes compared to recurrence rates of approximately 66–75% in those patients who have not undergone any of these therapies^22^. However, this information should be carefully analyzed since it is derived from a study with a small sample size (n=70) and with a short follow-up time after the therapy choice; therefore, it would be prudent to wait for further studies with a more refined methodology to reaffirm these inferences^22^.

The third theme is biliary sludge. Although the studies analyzed in this review did not indicate specific management for patients with biliary sludge associated or not with AC (this condition was considered an exclusion criterion in most of these studies), it was found that biliary sludge presence, even if associated with preserved functionality of the gallbladder, was regarded as risk factor for the occurrence of acute biliary events (such as biliary colic, cholecystitis, cholangitis, pancreatitis)^15^. A prospective study of 169 patients candidates for bariatric surgery evaluated in the preoperative period showed a 14.2% incidence of biliary sludge^20^. Subsequent to surgery, after 12 months of follow-up, it was found that 79% of these patients remained only with biliary sludge, 15.8% developed AC, and 5.2% developed symptomatic cholelithiasis^20^. It is also valid to present that in 31 patients (21.2%) who in the preoperative period presented no abnormalities in the gallbladder, after a follow-up of more than 12 months following bariatric surgery, some new biliary abnormalities were evidenced (18 cases of biliary sludge; 11 cases of AC; and 2 cases of symptomatic cholelithiasis)^20^. Thus, despite the low level of recommendation and evidence, in scenarios of patients with low preoperative surgical risk, one might indirectly infer that the management of biliary sludge might be similar to the reasoning for cases of biliary microlithiasis (IIIB)^15^.

As evidenced above, patients who undergo bariatric surgery should receive additional attention. Considering, classically, that this surgery is indicated for patients with obesity grade III (body mass index [BMI] >39.9) or grade II (BMI between 35 and 39.9) with comorbidities, the most frequent complication seen in the long term is the development of cholelithiasis, probably originated due to the significant weight loss that occurs during the postoperative period (especially those who lose more than 1.5 kilograms per week) associated with a higher excretion of cholesterol in the bile related to the rapid and important weight loss process^20^. Reinforcing this higher prevalence of cholelithiasis in patients after bariatric surgery, it is known that one-third will present with cholelithiasis or biliary sludge, in general, in the first 18 months after the procedure (IVC)^20^. This incidence may reach up to 53% of cases^20^. The same study observed that only the clinical follow-up (without prophylactic cholecystectomy) of patients who developed AC was safe since few of them became symptomatic (3.4%) or presented complications related to cholelithiasis in 12 months of follow-up after bariatric surgery (IVC)^20^. It is also valid to realize that in a prospective cohort of 959 patients who underwent bariatric surgery (92% were gastric bypass type with laparoscopic Roux-en-Y reconstruction), the rate of symptom development in the post-surgery period was 8% for the entire group (with or without prior AC) and 15% (IIB) for those with AC identified preoperatively^9^. Thus, we realize that AC before bariatric surgery is a risk factor for developing symptomatic cholelithiasis in the postoperative period (IIB)^9^. Although it has been observed that the prophylactic use of statins, alone or in combination with ursodeoxycholic acid, could reduce the formation of gallstones and biliary sludge in the period post-bariatric surgery, these prophylactic measures are not yet formally recommended (IIB)^9^; moreover, when used for direct therapeutic purposes for gallstone dissolution (with ursodeoxycholic acid) unsatisfactory results were obtained (dissolution of stones in 2.2% of the non-obese population tested) (IIB)^6^.

In contrast, a randomized trial with 222 patients recommended the concomitant performance of prophylactic cholecystectomy during bariatric surgery of the vertical gastrectomy type since it is a safe surgical procedure, even if the intraoperative and hospitalization times are longer (IIB)^10^. This indication was suggested in this study^10^ due to the high frequency of conversion to symptomatic cholelithiasis after bariatric surgery – about 55% of the group who did not have concomitant vertical gastrectomy became symptomatic and required late cholecystectomy (IIB)^10^. This high occurrence rate of AC symptoms was discordant with other studies addressing clinical follow-up after bariatric surgery and should be viewed cautiously^9,20^. In addition, this clinical trial^10^ also identified that weight loss percentage and family history were risk factors for developing symptomatic gallstones (IIB)^10^, likewise distinct from the other references^9,20^.

Furthermore, although laparoscopic cholecystectomy is considered a safe procedure, being related to a mortality rate of less than 0.2% and morbidity of less than 5.0%, other intrinsic risks should be considered^6^. It was identified that 10.8% of patients who underwent cholecystectomy presented complications within 30 days after surgery, the two most frequent being intra-abdominal collection formation and operative wound infection (IB)^1^.

Unfortunately, surgery may not guarantee the resolution of the preoperative clinical manifestations identified in the initial medical evaluation^1,6^. It has been reported that up to 40% of patients undergoing cholecystectomy have persistent pain or other abdominal symptoms – postcholecystectomy syndrome (abdominal pain, gastrointestinal disturbances, dyspepsia, heartburn, nausea, vomiting, jaundice, flatulence, persistent diarrhea or constipation) (IB)^1^ – drawing attention to the importance of identifying which patient with pain is associated with cholelithiasis, i.e., the one with a typical picture of biliary pain or the one that has evolved with a specific complication (choledocholithiasis, cholangitis, cholecystitis, and pancreatitis)^1^.

Table 4 summarizes the main advantages and disadvantages of surgical or conservative treatment (clinical follow-up) discussed above.

**Table 4:** Summary of advantages and disadvantages of surgical treatment (prophylactic cholecystectomy) compared to conservative treatment (clinical follow-up) in patients with asymptomatic cholelithiasis, according to the level of evidence.

Level of Evidence	Advantages of surgical treatment	Disadvantages of surgical treatment	Advantages of clinical follow-up	Disadvantages of clinical follow-up
I B	Prophylactic cholecystectomy during gastrectomies did not represent significant extra operative time and was associated with minimal additional risks^2^. Prophylactic cholecystectomy concomitant with oncologic gastrectomies had no significant impact on the natural history of cancer^2^.	The complication rate up to 30 days after cholecystectomy is 10.8%, the two main ones being reported as collection formation and infection of the operative wound^1^.	The natural course of the disease is benign and presents low lifetime mortality (less than 1% of people will die from gallstone-related causes)^1^.	About 20% of AC will become symptomatic in a mean follow-up time of 8.7 years, causing pain or complications (acute cholecystitis, empyema, choledocholithiasis, cholangitis, and pancreatitis)^1^.
II B	Laparoscopic cholecystectomy has a mortality rate of less than 0.2% and a morbidity rate of less than 5.0%^6^. Prophylactic cholecystectomy performed electively is associated with lower morbidity and mortality than when the surgery is emergency^6^.	After laparoscopic cholecystectomy, 9% of asymptomatic patients before surgery developed biliary-type pain after surgery; 27.3% of those with mild symptoms before surgery persisted with these symptoms after the procedure; 14.1% of patients with severe symptoms persisted with symptoms after surgery, although four of them evolved with decreased intensity^6^.	22% of previously asymptomatic patients developed symptoms during the follow-up period, but these disappeared in 58% and 52%, respectively, when they were mild and intense^6^. 1 to 4% of AC cases become symptomatic annually, reaching 20% in 20 years, and almost always had symptoms before a complication set in^21^.	–
III A	Prophylactic cholecystectomy after heart transplantation is recommended because it reduces mortality and costs, according to a *quality-adjusted life-year* assessment^4^.	Emergency cholecystectomy in post-transplant recipients is associated with significantly higher morbidity and mortality, respectively: 44 and 37% in heart transplant recipients; 33 and 5.6% in kidney transplant recipients^4^.	In cases of pancreas or kidney transplantation, the management of AC is recommended to be conservative since there has been no increase in AC-related morbidity in the post-transplant period^4^.	–
III B	–		–	During their lifetime, 25 to 33% of patients with AC develop symptoms or complications related to the disease^15^. In patients with AC, the rate of acute cholecystitis at 5-year follow-up was significantly higher in patients with coronary artery disease (CAD) (10.9%) compared to those without CAD (1.6%)^15^.
IV C	Asymptomatic biliary microlithiasis is present in 74% of cases of acute idiopathic pancreatitis, and prophylactic cholecystectomy would eliminate the possibility of recurrence of pancreatitis^22^.	Cholecystectomy concomitant to bariatric surgery had higher morbidity and mortality rates (higher occurrence of infections, pulmonary and gastrointestinal complications), more re-interventions, and longer hospital stays compared to bariatric surgery without cholecystectomy^20^.	Few patients with AC after bariatric surgery developed gallstone symptoms in the first 12 months of postoperative follow-up. The overall rate of cholecystectomy when the patient became symptomatic or developed a complication 12 months after bariatric surgery was 3.4%^20^.	–
V D	–	Cirrhotic patients have higher mortality rates when undergoing surgery and anesthesia (overall mortality rate of 11.6%) and even higher in those with Child-Pugh C liver function (mortality rate of 17%)^23^. The morbidity rate for cirrhotic patients after laparoscopic cholecystectomy is higher (21% of cases)^23^. Cirrhotic patients have a higher conversion rate from laparoscopic to open surgery^23^.		When symptoms appear, morbidity and mortality are higher in patients with cirrhosis compared to non-cirrhotic patients^23^.

AC: asymptomatic cholelithiasis.

Although the low mortality (<0.2%)^6^ and morbidity (5–10.8%)^1,6^ related to laparoscopic cholecystectomy, the evidence suggests that conservative treatment (clinical follow-up) for AC is feasible and safe. However, a significant portion of patients may become symptomatic over time (1–4% per year)^1,6^. Furthermore, the mortality risk associated with future gallstone-related complications is low (<1%)^1^, and these complications will almost always be preceded by typical biliary pain or other milder clinical manifestation^21^; and we also aim to prevent the dissatisfaction of cholecystectomized patients who may persist with symptoms or develop a postcholecystectomy syndrome^1^.

An exception should be considered for patients after heart transplantation (that it is associated with lower mortality)^4^ and in patients with biliary microlithiasis (stones <4 mm) or with biliary sludge of low surgical risk preoperatively, not cirrhotic^15,22^ or cirrhotic with preserved liver function^23^. Despite a lower level of evidence (IIIB), a possible surgical indication can still be reinforced, especially for those patients who present concomitantly with these conditions mentioned above, a coronary artery disease, since they have a greater chance of evolving with acute cholecystitis in 5 years of follow-up^15^.

## CONCLUSIONS

Most evidence points to the safety and feasibility of conservative treatment (clinical follow-up) of AC. However, in post-cardiac transplant patients and those with biliary microlithiasis with low preoperative surgical risk, a prophylactic cholecystectomy is recommended. However, more studies with better levels of evidence are needed to reinforce or refute the conclusions above, even due to the small sample size and follow-up time related to most of the analyzed studies.
